# Moral distress among neonatologists working in neonatal intensive care units in Greece: a qualitative study

**DOI:** 10.1186/s12887-023-03918-1

**Published:** 2023-03-08

**Authors:** Maria Deligianni, Polychronis Voultsos, Maria K. Tzitiridou-Chatzopoulou, Vasiliki Drosou-Agakidou, Vasileios Tarlatzis

**Affiliations:** 1 Laboratory of Forensic Medicine & Toxicology (Division: Medical Law and Ethics), School of Medicine, Faculty of Health Sciences, Aristotle University of Thessaloniki, University Campus, GR 54124 Thessaloniki, Greece; 2 Midwifery Department, School of Healthcare Sciences, University of Western Macedonia (Greece), Ikaron 3, GR 50100, Kozani, Greece; 3 1st Department of Neonatology, School of Medicine, Faculty of Health Sciences, Aristotle University of Thessaloniki, University Campus, GR 54124 Thessaloniki, Greece; 4 1st Department of Obstetrics and Gynaecology, School of Medicine, Faculty of Health Sciences, Aristotle University of Thessaloniki, University Campus, GR 54124 Thessaloniki, Greece

**Keywords:** Moral distress, Constraint distress, Uncertainty distress, Neonatologists, Neonatal Intensive Care Units (NICUs), Extremely Premature Infants (EPIs), Resuscitation decisions, Medical futility decisions

## Abstract

**Background:**

Working as a neonatologist in a neonatal intensive care unit (NICU) is stressful and involves ethically challenging situations. These situations may cause neonatologists to experience high levels of moral distress, especially in the context of caring for extremely premature infants (EPIs). In Greece, moral distress among neonatologists working in NICUs remains understudied and warrants further exploration.

**Methods:**

This prospective qualitative study was conducted from March to August 2022. A combination of purposive and snowball sampling was used and data were collected by semi-structured interviews with twenty neonatologists. Data were classified and analyzed by thematic analysis approach.

**Results:**

A variety of distinct themes and subthemes emerged from the analysis of the interview data. Neonatologists face moral uncertainty**.** Furthermore, they prioritize their traditional (Hippocratic) role as healers**.** Importantly, neonatologists seek third-party support for their decisions to reduce their decision uncertainty. In addition, based on the analysis of the interview data, multiple predisposing factors that foster and facilitate neonatologists’ moral distress emerged, as did multiple predisposing factors that are sometimes associated with neonatologists’ constraint distress and sometimes associated with their uncertainty distress. The predisposing factors that foster and facilitate neonatologists’ moral distress thus identified include the lack of previous experience on the part of neonatologists, the lack of clear and adequate clinical practice guidelines/recommendations/protocols, the scarcity of health care resources, the fact that in the context of neonatology, the infant’s best interest and quality of life are difficult to identify, and the need to make decisions in a short time frame. NICU directors, neonatologists’ colleagues working in the same NICU and parental wishes and attitudes were identified as predisposing factors that are sometimes associated with neonatologists’ constraint distress and sometimes associated with their uncertainty distress. Ultimately, neonatologists become more resistant to moral distress over time.

**Conclusions:**

We concluded that neonatologists’ moral distress should be conceptualized in the broad sense of the term and is closely associated with multiple predisposing factors. Such distress is greatly affected by interpersonal relationships. A variety of distinct themes and subthemes were identified, which, for the most part, were consistent with the findings of previous research. However, we identified some nuances that are of practical importance. The results of this study may serve as a starting point for future research.

## Introduction

The distinct concept of moral distress in the clinical context was first defined by Jameton in 1984 as “the psychological distress of being in a situation in which one is constrained from acting on what one knows to be right” [1, 2]. This definition of moral distress in the clinical context is narrow and focuses on the occurrence of such distress in “situations in which a person is constrained from taking the correct action, as some obstacle (e.g., an institutional rule or a physician’s decision) stands in the person’s way” [3]. In that context, Wilson et al. recently noted that “moral distress refers to the emotional experience of feeling involuntarily complicit in an unethical act but have little power to act differently or change the situation” [4]. This discrepancy inherently challenges the ethical principles and values of the person who must take an action that is contrary to what he or she believes is the appropriate care plan. Importantly, this person “knows and believes what is morally right” [5]. However, he or she has “no choice but to act this way” [6]. This situation is referred to as “constraint distress” [7]. Nevertheless, the definition of moral distress has been revised and expanded in recent years. According to a broader definition, moral distress can emerge in morally undesirable situations other than those that involve constraints [7]. In contrast to the narrow definition provided previously, this type of distress is commonly referred to as “uncertainty distress” [3]. Many key ethical dilemmas that occur in clinical practice are complex and difficult and involve high levels of moral and scientific uncertainty regarding prognosis, which can give rise to considerable uncertainty distress [8]. The concept of constraint is central to the original (strict) definition of moral distress, whereas the concept of uncertainty is central to the newer (broader) definition of moral distress [3].

With regard to neonatal health care, it has been argued that “moral distress is prevalent in the neonatal intensive care unit (NICU)” and that such distress is “far more prevalent in the clinical context than even the current literature describes” [9]. Working as a neonatologist involves ethically challenging situations and dilemmas that must be solved against a backdrop of great uncertainty in the context of neonatal intensive care units (NICUs). In the context of the NICU, health care providers confront morally challenging situations and are involved in medically complex decision-making in conditions of uncertainty on a regular basis. The NICU is a high-tech and stressful setting in which “decisions regarding end-of-life care, periviable resuscitation, and medical futility are common” [9]. Neonatologists must make difficult ethical decisions in the context of caring for critically ill infants [10]. Indeed, in the context of the NICU, ethical challenges are frequently raised, which at times becomes tragic, especially with regard to providing care for infants who are born at the limit of viability (weeks 22–25 of gestation). Extremely premature infants (EPIs) are infants who are born at an extremely low gestational age (GA), that is, < 26 weeks [11]. In cases that concern the limit of viability (i.e., the “grey zone”), the conflict among ethical principles becomes extremely great, and many ethical questions remain without definite and undeniable answers. Making resuscitation decisions for EPIs or treatment decisions for infants in the NICU is a difficult task that can often give rise to moral distress [8]. Neonatologists’ resuscitation decisions involve an attempt to identify the infant’s best interest and strike a balance between the infant’s best interest and respect for parents' autonomy. This decision is difficult and complex, and it is becoming increasingly difficult because preterm birth and survival rates at lower gestational ages are increasing worldwide [12, 13]. Making resuscitation or care decisions for infants born at very low gestational age involves great prognostic uncertainty [8, 14]. Therefore, such decisions often give rise to uncertainty distress. Uncertainty is a key contextual factor that significantly affects decision-making. Furthermore, no “best available evidence”-informed guidelines are presently available, while existing guidelines are inadequate to address neonatologists’ needs [8, 13, 15–17].

While uncertainty or moral dilemmas do not automatically result in moral distress, these situations can lead to moral distress among neonatologists if their core values are challenged and their moral integrity is compromised [9]. Neonatologists are often involved in situations that place them at odds with their own concepts of appropriate treatment and their own values and beliefs.

Moreover, neonatologists may feel constrained from pursuing what they believe to be the right course of action with regard to treating their child’s illness due to external (i.e., institutional or financial) or internal constraints. Internal constraints may be the result of an empathy-driven emotional response or the neonatologist’s wish to be a good physician who works to fulfil his or duty duty, which may serve as a strong internal motivator to take an action that conflicts with what he or she believes is the appropriate care plan. Even when neonatologists have made a clear judgement regarding what course of action should be taken, they may feel as if they are prevented from acting accordingly. This constraint may compromise their moral integrity and give rise to constraint distress, namely, moral distress in the strict sense of the term [1, 2].

In the extant literature, it has been argued that neonatologists’ moral distress is a topic that continues to warrant further exploration [8]. The vast majority of studies on the neonatal resuscitation decisions made by neonatologists have employed quantitative methodologies, which provide little insight into how neonatologists make or perceive their decisions. The few qualitative studies that have been conducted in this context have focused primarily on the views of other stakeholders involved in the shared decision-making process (e.g., parents, other physicians or nursing practitioners) rather than on the views of neonatologists [8, 16]. These few qualitative studies have identified parental involvement and great uncertainty as the key factors that make it difficult for neonatologists to make decisions regarding the resuscitation of extremely premature infants [8, 16]. In summary, few studies have explored the concept of moral distress in the stressful environment of neonatal intensive care units. The aim of this qualitative study was to explore how neonatologists experience moral distress in the Greek health care context in further detail. To accomplish this goal, we used neonatologists’ narratives to develop a deep and nuanced understanding of concepts of moral distress among neonatologists. To the best of our knowledge, in Greece, there are 30 NICUs (20 in the public health care sector and 10 in the private health care sector). Approximately 180 neonatologists work in these NICUs. For the purpose of this study, moral distress in the strict sense of the term and moral distress in the broad sense of the term are hereafter referred to as constraint distress and uncertainty distress, respectively.

### Research questions

This research sought to answer the two following questions that delineate the focus of this study:a) What experiences with feeling “prevented from acting on what you knew to be right” did neonatologists who had been working in Greek neonatal intensive care units (NICUs) for at least one year before the interview took place have?b) What experiences did the abovementioned neonatologists have with high-risk decisions in situations involving uncertainty regarding the infant’s prognosis or the appropriate treatment or diagnostic intervention?

## Methods

### Study design

The present work was a prospective qualitative research study based on in-depth interviews conducted with neonatologists who had worked in Greek neonatal intensive care units (NICUs) for at least one year prior to the interview. This qualitative descriptive study was conducted from March 2022 to August 2022. Thematic analysis was selected as the methodological orientation of the study.

### Participants

#### Sampling and data collection

To obtain a wide range of perspectives, a combination of purposive and snowball sampling was used. First, purposive sampling was used to select participants who met the eligibility criteria discussed below. The participants were drawn from one NICU in the region of Thessaloniki (*n* = 12). Thereafter, we employed snowball sampling by asking informants to refer other potential participants with the aim of including more participants in the sample (*n* = 8). None of the early informants had already been selected through the previously employed purposive sampling. These informants included researchers’ personal acquaintances. While the diversity of samples generated using the snowball sampling method is questionable, we attempted to enhance the sample diversity to broaden the range of participants’ viewpoints to the greatest extent possible. As “sample seed diversity is important to achieving sample diversity”, we attempted to ensure that the initial set of informants in the snowballing process exhibited notable variety. Moreover, to ensure “chain referral sampling” to the greatest extent possible, every “reach-out” was “carefully tracked and followed by a reminder” [18]. Ultimately, we obtained a sample including participants who exhibited variation in terms of age, gender and clinical experience and who were drawn from different NICUs throughout the whole country. None of the participants were already acquaintances of the interviewer.

#### Inclusion/exclusion criteria

The inclusion criteria for participation in the study were (1) being a neonatologist currently working in a Greek NICU and (2) having worked in a Greek NICU for at least one year prior to the interview. The exclusion criterion for participation in the study was the inability to communicate effectively in the Greek language.

### Data collection

#### Interviews

The one-on-one interviews were conducted between February 2022 and August 2022 and lasted between 46 and 59 min each. The mean length of the interviews was 51 min. The interviews were audio recorded and transcribed verbatim into Greek*.* Relevant field notes were written both before and after the interviews by the interviewer to help produce a comprehensive set of insightful findings. The language used in the interviews was Greek. The interviews were held at interviewees’ preferred times in quiet and neutral places of the participant’s choice, specifically in comfortable environments.

#### Interview guide

An interview guide was developed based on a review of the relevant literature [1–3, 6–10, 13, 15–18]. To obtain a deep and comprehensive understanding of the concepts under investigation (neonatologists’ moral distress), the interviews covered a number of topics intended to capture a wide range of the participants’ lived experiences. Below, we specify a few questions that were included in the interview guide:

- 1) If you have ever face the question of whether the treatment you provided or recommended was the right one, how did you respond to these situations? Why did you respond as you did?
- 2) Please describe to me in detail any significant difficulties you encountered in making a clear judgement regarding what course of action should be taken.
- a) Please describe to me in detail real past case(s) (if any) in which you felt constrained from acting on what you knew to be right. Please describe to me in detail your related experiences.

### Interviewer-interviewee relationship

The interviewer attempted to build trust with interviewees before the interview itself and to ensure that the interviewees were comfortable. The interviewer made great effort to prevent casual questions from becoming questions that might exacerbate unconscious interviewer bias. She made every effort not to ask leading questions. While the interviewer is a paediatrician, she remained neutral on the issues that were discussed with the neonatologist. As a phenomenological researcher, she maintained an unreflective and effortless (normal) attitude. Reflexive thinking was used throughout the research process to reduce unintentional personal bias. Participants were asked broad questions and prompted to expand upon the issues that they considered to be most relevant.

### Data analysis

Data collection ceased only when data saturation was reached. Thus, data collection continued through 17 interviews. Three additional interviews were conducted to ensure data saturation. A thematic analysis of the data was performed [19]. All the coauthors contributed to the analysis. They engaged with one another to limit research bias. The researchers made every effort to ensure the validity and reliability of the study based on Gibbs’s recommendations [20]. Validity was enhanced by using maximum variance in participant selection.

After each interview was transcribed verbatim, the interview data were analysed by carefully reading and rereading each interview transcript to obtain a good overall sense of the whole interview [19]. Subsequently, units expressing meaning were identified in each interview transcript, and units that were similar in meaning were coded accordingly. The researchers compared the data to ensure consistency among the codes. Codes with similar meanings were grouped into subcategories. These subcategories were then condensed into broader categories. The categories were grouped into prevailing themes as the final product of the analysis. Disagreements among the authors were addressed through further discussion.

Data analysis was conducted using NVivo qualitative data analysis software version 9, which was released in 2010.

### Ethical considerations

Written informed consent was obtained from the participants*.* If neonatologists were willing to participate, they were provided with adequate information regarding the design, purpose, nature and confidentiality of the study, including the facts that their participation was voluntary and that their consent could be withdrawn at any time during the course of the study without any reason or reprisal. The importance of maintaining anonymity and confidentiality was emphasized. Anonymity and confidentiality were ensured throughout the study. Interview data were anonymized during transcription. To preserve the participants’ anonymity, no names are used in this paper, and numbers (e.g., P1) are used instead. The interviews were registered and stored in a strictly confidential fashion. The study and consent procedures were approved by the Ethics Committee affiliated with Aristotle University of Thessaloniki, Faculty of Health Sciences, School of Medicine (Decision Number: 2.437/24–11-20). All methods were performed in accordance with the relevant guidelines and regulations.

## Results

The participant characteristics are presented analytically in Table 1.

**Table 1:** Participant characteristics

Participant	Work experience (years)	Gender	Age (years)
P1	40	Female	65
P2	7	Female	40
P3	13	Female	49
P4	2	Male	41
P5	3	Male	42
P6	1,5	Female	33
P7	1	Female	38
P8	15	Female	55
P9	4	Female	44
P10	1	Female	33
P11	7	Female	39
P12	7	Male	38
P13	3	Female	40
P14	10	Male	51
P15	1	Female	47
P16	2	Female	36
P17	1,5	Male	35
P18	4	Female	40
P19	1,5	Female	35
P20	1,5	Male	35

The thematic data analysis revealed five major themes and subthemes (Table 2).

**Table 2:** Major themes and subthemes

Theme	Subtheme
1. **Neonatologists are confronted with moral uncertainty** (which in turn causes moral distress)	
2. **Neonatologists prioritize their traditional (Hippocratic) role as healers**	
3. **Neonatologists seek third-party support for their decisions to reduce their decision uncertainty**	
4. **Multiple predisposing factors foster and facilitate neonatologists’ moral distress**	**4.1. Previous experience is important to mitigate neonatologist’s moral distress** 4.2. **The lack of clear and adequate clinical practice guidelines/recommendations/protocols** 4.3. **The scarcity of health care resources** 4.4. **The fact that in the context of neonatology, the best interest and quality of life of the infant are difficult to determine** 4.5. **Making decisions in a short time frame**
5. **Multiple predisposing factors are sometimes associated with neonatologists’ constraint distress and sometimes associated with their uncertainty distress**	5.1. **NICU directors** 5.2.** Colleagues working in the same NICU** 5.3.** Parents’ attitudes**
6. **Neonatologists become more resistant to moral distress over time**	

### Neonatologists are confronted with moral uncertainty (which in turn causes moral distress)

Neonatologists are often confronted with ethical dilemmas that involve conflicting obligations that are perceived as irreconcilable; that is, they face tough situations in which they have two moral obligations, which cannot be met simultaneously or within the available time frame and the current circumstances. These obligations are based on conflicting values against a backdrop of high prognostic uncertainty. Neonatologists must face conflicting obligations that are perceived as irreconcilable after every effort is made to make the best of all the currently available sources of support for their decision. Nevertheless, neonatologists are obliged to act on their decisions even when facing irreconcilable dilemmas. This situation causes them to experience the psychological impact of intense internal moral conflict, that is, what we call moral schism, which refers to the need to choose between providing resuscitation for an EPI who has a slim chance of living a long life without experiencing neurodevelopmental disorders and allow the infant to die to prevent a life with very low quality of life (Q-o-L), whatever “low Q-o-L” means to family and society. The following quotations illustrate this point:

In such a vague case, like the one described before, which is a baby’s resuscitation, how long can one go on? How long can one prolong survival? One, maybe two hours? I don’t know if all of this makes any sense. It’s such a stressful situation. Many times, I keep asking myself: What did I actually achieve, after all? The quality of life is such a big issue. No matter how much the weight is, 500 gr or even 450 gr, I can still resuscitate the baby, get it connected to the machine and support breathing through the machine. And then what? And again, we hear of cases, very rare ones in terms of the literature indeed, according to which babies were resuscitated and had good progress. The percentage is, of course, very small. But in the end, is it worthwhile to try even for this one in ten thousand percent? I don’t know, I cannot have a clear view on such a matter (P3).

Similarly, participants made the following statements: *Is this such a case as well, 1 in 1000, that will eventually survive?* (P13)… *is it the 1%* … (P2, P8, P12). *Miracles do happen sometimes* (P2, P8, P12, P20) … *complicated things, ok* (P20). *But what if the miracle happens after all? This is surely excruciating. The uncertainty is extreme, not only about the baby’s survival but about what is going to happen in the future generally* (P17) … *should you be interested in the survival itself, or is quality of life important as well? You are surely interested in both factors* (P18). *I may have a child that is alive, but what kind of child will I eventually deliver to the parents and to society?* (P3) … *what if, in the end, the baby does survive but has numerous neurological deficits/disorders?* (P19). *The most important thing for me is the baby’s neurological development…. Whether it will suffer from heavy brain damage that will affect its whole life, the family’s life and may have an impact on society as well* (P6). *I have had the experience of parents coming to our offices and referring to the issue indirectly, saying that the brain damage/lesions is/are too severe. We didn’t want you to try so hard* (P9). *Who is ready to take care of a child with possible deficits? What social system can support such a child? What social structures?* (P14). Indeed, participants felt that prognostic uncertainty regarding the alleviation of long-term suffering (e.g., pain or disability) for infants and families may give rise to uncertainty distress, especially given that Greece is a country that features a suboptimal social welfare program for disabled individuals (P14).

Theory has not yet produced (and in all likelihood cannot produce) clear guideline-based resuscitation thresholds. Participants P11, P12, P14 and P17 noted that some directions have been made available, but these guidelines do not cover all cases. One must use his or her own judgement: *There are certainly protocols in use, but you always put your own factor and your own personal question mark. Should I try a bit more? Should I not let it go? What if? What if?* (P17).

Furthermore, it should be noted that while the fear of lawsuits or being sued by parents plays a key role in this context, the main reason for neonatologists’ feelings of uncertainty seems to be rooted in ethics and based on their professional responsibility. This conclusion was drawn from the considerable emphasis that participants placed on the need to compromise their moral values.

### Neonatologists prioritize their traditional (Hippocratic) role as healers

With even the slightest chance of succeeding, neonatologists remain motivated and continue to provide care. If an infant has even the slightest chance of living a long life without experiencing neurodevelopmental disorders, that is, without significant suffering or disability, participants noted that they would not give up fighting for it, even if doing so conflicted with the current guidelines. Clinical practice guidelines/protocols were perceived as strictly necessary but insufficient for making resuscitation decisions. Participants prioritized their traditional (Hippocratic) role as healers. That is, they seemed to be intuitively predisposed towards maximizing benefits and minimizing harms due to their developed professional conscience and to be strongly committed to their values, i.e., to what they understood to be the core purpose of medicine and the role of the healer. This theme was a recurring finding in almost all interviews. More precisely, neonatologists noted that they would choose to provide a treatment that offered the possibility of a “miracle”. Several participants noted that they had rarely been involved in situations in which a newborn with the slightest chance of living a long life without significant suffering or disability changed unexpectedly for the better, whereas they had experienced situations in which babies with very high chances of living a long life without significant suffering or disability experienced an unexpected decline and died. They noted that they would withdraw the treatment or would not resuscitate if and only if there were not even the slightest (extremely low, i.e., according to participants “ < 1%”) fighting chance, such as in the case of a chromosomal abnormality (e.g., P1, P11, P17, P20). The following quotation illustrates this point: *I, personally try in 99.9% of cases; I do whatever I can, I do my best, unless there is, as I mentioned before, some very severe brain damage or some chromosomal abnormality or a heavy syndrome that is incompatible with life* (P1). Fortunately, neonatologists working in NICUs in Greece do not face such tough ethical dilemmas particularly frequently. According to the participants in this study, each of them had to deal with such difficult decisions approximately 2–4 times per year, namely, approximately 10–40 times over the past five years.

Furthermore, participants in this study admitted that while modern guidelines and protocols incorporate a great deal of experience from colleagues worldwide, they do not cover every case. They noted that a neonatologist must make decisions based on his or her own personal judgement and evaluation (P11, P14, P17). Participants highlighted their reluctance to continue providing life-sustaining treatment that would cause infants to experience significant suffering (P14, P15, P16, P17). They emphasized the fact that life-prolonging treatment may cause newborns to feel physical pain and suffering (e.g., due to intubation, venipunctures, parenteral nutrition) in addition to the amount of pain and suffering that they are already experiencing. Participants emphasized the fact that the balance between benefits and harms or burdens in a particular case should be evaluated as accurately as possible from the neonatologist’s perspective …*so as not to cause extra pain to the baby and forlorn hope for the parents* (P17). A finding revealed in almost all interviews was that participants declared that on their view, there is no such thing as “futile care” and that the term futility has no utility and should be abandoned by neonatologists. Participants (P3, P10, P19) made the following statements: *I think it is worth the effort to try and save the infant’s life, eventually, which shows how things will end up* (P19). *[Besides,] clinical experience has shown that sometimes cases that are considered doomed and treatments that are considered futile might not be so after all* (P11).

### Neonatologists seek third-party support for their decisions to reduce their decision uncertainty

Neonatologists feel professionally and personally vulnerable in the context of ethical decision-making. When considering difficult ethical dilemmas, they experience high levels of decision uncertainty for ethical reasons rather than legal reasons (i.e., fear of legal consequences). Neonatologists seem to have a strong sense of moral responsibility. Neonatologists “allow” themselves the opportunity to make their own ethical decisions. Furthermore, they need their director or colleagues to stand by them through the process of making tough ethical decisions. All participants strongly expressed the view that even seemingly simple decisions may warrant a second opinion. Based on our data analysis, we found that neonatologists do not feel as if they are isolated health professionals but rather as if they are surrounded by supportive colleagues or other key stakeholders who are involved in shared decision-making processes (e.g., infants’ parents).

All participants mentioned their need for approval of and support for their personal decision from their colleagues to experience less decision uncertainty. Participants noted that they would seek a second (supportive) opinion to mitigate their decision uncertainty. Therefore, they make every effort to take advantage of all the most helpful and currently available sources of support. In that regard, they consult existing guidelines and protocols, draw from their own previous experience, seek out second (supportive) opinions from highly expert sources, that is, their director or colleagues, especially those with considerable previous experience, to inform their decisions and address their decision uncertainty: *…there, you rely on the patient’s clinical picture, you use your own scientific training, you ask for experience and knowledge from someone more experienced than you or someone who is above you in the hierarchy. You search the bibliography for similar cases, you ask for opinions, and I think that, in this way, you can diminish uncertainty to a great extent* (P2) … *we try to act based on previous experience and protocols, we seek the opinion of other—especially older—colleagues and the clinical director himself, keeping the parents updated at the same time … I consider communication among colleagues to be extremely important … I will trust the opinion of both a colleague who is my superior and that of the clinical director. I want to discuss it. The best thing, in my opinion, is when the case is discussed with the whole team* (P10) …. *my conscience is unbearably stressed, I have to share it with others, I have to listen to what my colleagues have to say, to talk to my director, I search the bibliography and, last but not least, I act while keeping in mind the values and beliefs of the parents that I have before me* (P5). *My decision is influenced by my colleagues and the director. It is a dynamic situation that is developing* (P13) … *I will surely ask for help from my superiors and my director* (P20).

In addition, neonatologists seek the approval and trust of the infants’ parents. Parental approval of the neonatologists’ decisions reduces their uncertainty. Participants P9 and P13 noted that they would appreciate greater parental involvement with responsibility. They expressed their strong desire to share the burden of ethical responsibility with the infant’s parents. Participants emphasized their need to be familiar with parents’ values and preferences (P9, P12). Participant P9 made the following statement: *Look … If the parents, despite the newborn’s problems, say that they want to keep the baby because this is God’s or fate’s decision, I will do my best. If, on the other hand, they say that they cannot carry the burden, I respect it, I understand. I will still do whatever I can, but I won’t push things. I won’t intervene in an extreme or aggressive way … when I see the doubt in the parents’ eyes, I am under extreme psychological pressure …*

Furthermore, neonatologists seek advice from ethical committees and experts, which, in the participants’ opinion, should be established in every hospital (e.g., P15). Moreover, participants in our study expressed their desire for clearer guidelines and protocols, as is the case with respect to guidelines and protocols in some (though not all) other countries (e.g., the UK) (P17).

## Multiple predisposing factors foster and facilitate neonatologists’ moral distress

### Previous experience is important to mitigate neonatologists’ moral distress

Neonatologists explore their own previous experience with similar dilemmas in NICU clinical practice. Participants noted that their previous experience with similar ethical dilemmas in NICU clinical practice contributed to the mitigation of the decision uncertainty distress that they experienced (P1, P2, P3, P4, P5, P7, P9, P10, P17, P20).

### The lack of clear and adequate clinical practice guidelines/recommendations/protocols

Clear and adequate guidelines/recommendations/protocols contribute to the mitigation of uncertainty distress. Participants expressed feelings of intense uncertainty because in Greece features a regulatory gap in this context, and the existing regulatory framework includes many vague points (P1, P17, P19).

### The scarcity of health care resources

The scarcity of health care resources (whether human or material) may be an organizational/financial reason for the emergence of neonatologists’ moral distress. Participants P1, P3 and P5 emphasized the fact that neonatologists experience distress because of the lack of human and material resources: … *in a short time, I am supposed to take care of one or two newborns while I’m on call, for example, without any help. The available staff is limited, and the means are sometimes also limited. But one has to deal with the case on the spot. There’s no time to think and react. The right decision has to be made instantly* (P5). The moral distress described in this quotation is a combination of constraint and uncertainty distress. However, it is more closely related to constraint distress than to uncertainty distress.

### The fact that in the context of neonatology, the best interest and quality of life of the infant are difficult to determine

Another source of uncertainty distress is the fact that the terms “infant’s quality of life” and “infant’s best interest” are not only vaguely defined but also scarcely determined, as is the notion of the right “thing to do” in the context of neonatology (P17, P19). The ambiguous definitions of these notions and the corresponding great prognostic uncertainty could explain why participants in this study perceived conflicts among fundamental bioethical principles to be extremely challenging or irreconcilable and implied that lack of resolution caused them to face high risks of uncertainty distress.

*We add pain and…. pain to the newborn and burden to the parents. And for what reason? Is what I’m doing in favour of the baby?* (P4)… A similar claim was made by Participant P2. Participants P6, P11 and P12 felt uncertainty distress due to uncertainty regarding whether they had done the right thing. As Participant P13 asked, *What do I actually offer to the baby? In other words, who am I trying to help in the end? The baby? I’m not helping it. Me? So that I feel that I did something? The parents? To show that I’m exerting myself? I don’t know.* Furthermore, Participant 13 noted that *the fact that I put a newborn through such an ordeal to simply show that I simply tried makes me feel awfully stressed* (P13). A similar claim was made by Participant P14, who asked *How is it possible to hit a newborn continually and catheterize it at the very moment when it is dying? Am I doing that in order to prolong life for 1, 2, 3 days?* Interestingly, participants made the following statement: *You keep wondering if you are doing the right thing … Of course, somebody may pose the question: what exactly is the right thing, and who defines the right thing? And this is a big bioethical, philosophical discussion* (P17). *Maybe we should define once again what we mean when we refer to the quality of life. There are parents who tell us that they want this kid. It will be in a wheelchair, but we will offer it all the love and devotion it deserves because it is a human being, it has a personality* (P19).

In that regard, one participant explicitly mentioned experiencing difficulty striking a balance between the infant’s best interest and respect for parents' values and preferences: *The effort to ensure balance puts me under extreme stress. On the one hand, I need to act in favour of the newborn, and on the other hand, I should be able to support what the parents believe and what is good for them, up to a point, of course …* (P18).

### Making decisions in a short time frame

The notion of a short time frame emerged from the data analysis as a key predisposing factor associated with intense uncertainty distress among neonatologists. To make the best possible decision, neonatologists must be provided with correct and sufficient information or support by third parties. However, making decisions in a very short time frame prevents neonatologists from mitigating their decision uncertainty by obtaining reliable third-party approval. Obtaining such approval or support becomes an unattainable task if the decision in question must be made in a short time frame, as in the case of a neonatologist who is involved in a periviable childbirth and must make resuscitation decisions in a short time frame in the delivery room in the absence of any specific and clear guidelines-based resuscitation threshold: *If a delivery of a very premature baby occurs, there is no time to discuss anything or to refer to the bibliography. You are pressed for time to make a decision. And of course, the outcome of this case is uncertain. It’s questionable whether you have a few seconds or even a minute to listen to the parents’ opinion or inform them of what is going to happen. That is uncertainty* (P13).

Participants P12 and P16 made similar claims, as did Participants P5 and P9, who referred to the case of a neonatologist who must prioritize patient care plans for multiple infants during a shift, in which context the neonatologist must evaluate whether it would be worthwhile to invest a great deal of effort and consume his or her energy in caring for an infant who has the slightest chance of living a long life without experiencing neurodevelopmental disorders or in caring for another infant who has who has good chance to live a long life without experiencing neurodevelopmental disorders: …..*you may use your energy on a case which is doomed, while next to you there are another two, three, four newborns that have very good prognoses* (P4).

These circumstances make it difficult for the neonatologist to obtain a second supportive opinion from a reliable source. In such situations, the neonatologist is forced to make a decision based on practical wisdom (i.e., the notion of Aristotelean phronesis) and his or her own conscience; accordingly, his or her uncertainty distress is very high. In addition, the data analysis revealed that neonatologists’ constraint distress may be higher during regular working hours than during their shifts. Conversely, neonatologists’ uncertainty distress may be higher during their shifts than during regular working hours.

Furthermore, to bring our discussion of this theme (4) to a close, it should be noted that limited evidence of the effectiveness of a certain medication with regard to neonates increases neonatologist’s uncertainty distress. When a neonatologist must administer medications for “off-label” indications in NICU clinical practice (i.e., when treating a premature infant), uncertainty distress becomes increasingly important (P11).

## Multiple predisposing factors are sometimes associated with neonatologists’ constraint distress and sometimes associated with their uncertainty distress

Another finding that emerged from data analysis was that key predisposing factors associated with uncertainty distress among neonatologists might also serve as key predisposing factors associated with constraint distress among neonatologists and vice versa. NICU directors, neonatologists’ colleagues or hospitalized infants’ parents may serve as constraints or factors that cause decision uncertainty to remain high (such as when neonatologists perceive their decision-making support as lacking or inadequate). Furthermore, it should be noted that factors such as the NICU director, colleagues and parents may serve not only as direct constraints but also as indirect constraints: *There is, of course, the director, there are the parents, there are the other colleagues to whom you are accountable, literally or metaphorically* (P10).

### NICU directors

The vast majority of participants noted that the NICU director is perceived as an “orchestra director”, i.e., as responsible for a good mood and communication among health care workers in the NICU and as the person who suggests the “optimal” solution or recommendation in cases of tough situations that involve difficult ethical dilemmas. The director of the NICU may support the decision of a neonatologist working in the NICU. He or she may be the most reliable source of support. NICU directors who do not take a clear stance on an infant’s treatment do not contribute to mitigating the neonatologists’ decision uncertainty, namely, such directors do not reduce neonatologists’ decision uncertainty. Unsurprisingly, while participants working in the NICU did not feeling forced to comply with their directors’ recommendations, most participants noted that they would show respect for hierarchy with the aim of mitigating their decision uncertainty. As a result, they felt that they were prevented from acting on what they knew to be right. Participants always mentioned this point in cases in which they felt “forced” to continue providing care for an infant despite the fact that their own view was completely in opposition to continued care. The data analysis indicated that NICU directors represent a key predisposing factor associated with intense constraint distress among neonatologists. However, several participants declared that they tended to prioritize their own values and beliefs over those of other stakeholders involved in shared decision-making, i.e., NICU directors, colleagues, and parents. For instance, despite the director’s recommendation to stop a treatment that – in the director’s opinion – may cause both baby and neonatologists to suffer a great deal for no reason, some participants continued providing life-sustaining treatment, that is, they continued to fight for the slightest chance of success. Participants always mentioned this point in cases in which they felt “forced” to cease providing care to an infant despite the fact that their own view was completely in opposition to this decision. None of the participants claimed that they had stopped or would stop providing care for an infant when their NICU director had expressed the opposite view. However, they described their intense moral (constraint) distress in this context:

*I am in conflict with my personal beliefs, and I am stressed and psychologically overwhelmed when I feel that the right thing to do for the newborn is something else, that I am intervening and torturing the baby while there is no hope, but eventually I am pushed by other factors, such as the administration or the clinic protocol or the parents, to act in another way* (P15). Participants P14, P16, P19, and P17 made similar claims, adding that …*there I feel extremely pressed because I think that what I’m doing to the baby is not only meaningless but also harmful and bad* (P17). Participant P3 described her experience of constraint distress associated with the NICU director. As she noted, *The director has the final word. I may have a different opinion, but I have to follow and respect the hierarchy. I remember a case of hospital discharge many years ago for which I was facing a lot of pressure, as the newborn had to leave the intensive care unit to avoid having too many babies in the unit because there were no more beds available. This specific baby was not properly checked, in my opinion* (P3).

Nevertheless, Participant P8 mentioned a situation in which she acted on her refusal to comply with the requests and recommendations of her NICU director: *Now, for example, the director says that in a certain case, we shouldn’t intervene aggressively because it is a hopeless case, incompatible with life, and the baby will die, so we shouldn’t lose energy, neither the doctor nor the baby. Yes, there’s where I have a problem. Yes. There, I act according to my values. In such a case, I would do what I believe is right. Anybody can have their opinion, but we did not graduate together, nor did we take an oath together, and nobody can tell me that their beliefs are superior to mine. In such a case, I will do what I believe is right…. I had a baby have blood transfusion in secret once. I don’t consider it ethical for a baby to die of anaemia* (P8). Similarly, another participant noted that *I proceed according to my values and beliefs; therefore, my values and beliefs…. free me, for I know that I acted according to my conscience, and that will not lead me to a moral dead end, I will sleep tighter, to put it this way* (P9).

### Neonatologists’ colleagues working in the same NICU

The data analysis indicated that neonatologists’ colleagues working in the same NICU represent a key predisposing factor associated with uncertainty or constraint distress among neonatologists. Participants noted that they felt a strong need for their colleagues’ approval or at least their colleagues’ support (not necessarily unequivocal support) when making a difficult decision regarding an infant’s resuscitation or care. They feel much better when their colleagues support their choices or at least do not make negative comments or say “I told you so” after the events in question. In that regard, good and effective communication among NICU staff members was reported to be a highly important factor for boosting neonatologists’ moods and enabling them to remain motivated at work and to avoid experiencing high levels of uncertainty.

Organizational problems in the NICU, such as poor relationships and ineffective communication among colleagues in the NICU, may also be important predisposing factors in the development of uncertainty distress. While a neonatologist’s colleagues who have previous experience with similar ethical dilemmas in clinical practice may support the decisions of that neonatologist, thus mitigating his or her uncertainty distress, these colleagues may also serve as constraints that prevent a neonatologist from acting on what he or she knows to be right; that is to say, such colleagues may cause them to experience constraint distress. To illustrate this point, we cite a quotation from the interview with Participant 13. As she said, …*when I see doubt or a lack of acknowledgement in the eyes of my colleagues….. I feel the need for cooperation and acknowledgement from my colleagues, not doubt. I need them to help me when I face a dilemma.*

Furthermore, participants in this study developed constraint distress because in discussions between the staff members working at a NICU, they expressed one view, while their colleagues expressed a completely contrary view. Indeed, some participants’ colleagues insisted on continuing the treatment in contrast to the existing guidelines for the sake of appearances (P14). As participants felt morally obliged to show respect for the good relationships and effective communication among NICU staff members, they felt as if they were prevented from acting on what they knew to be right. Participant P14 described his experience of constraint distress associated with his colleagues as follows: *“Another factor of moral distress is when I don’t agree with my colleagues as to what we should do. That’s the worst… not simply to disagree, but to face a response, and in the end the prevailing view is not that of common sense or the protocol itself but rather the fact that we need to try for the sake of appearances. How should I put it differently? That’s what makes me wonder if we are really doing the right thing. I cannot get over it easily* (P14).

Furthermore, all participants strongly supported the view that good cooperation and good organization within the NICU, decision support from colleagues and a lack of colleagues’ concerns regarding whether the decision made was correct are prominent factors associated with reductions in uncertainty distress:

To mitigate their feelings of uncertainty, participants highlighted the need for a *good collaboration atmosphere* (P1, P13), *organization, unity, and unanimity in the NICU* (P2, P13). Participants P19 and P20 explicitly highlighted the fact that the NICU director is a person who is committed to ensuring and promoting harmony and cooperation in the NICU as a workplace: *The director plays an important role because he directs the whole situation. He sets the tone* (P19). *The director must lay the groundwork for collaboration.* (P20). As Participant P13 said, *I feel psychologically stressed when I see doubt in my colleagues’ eyes. I need their help when I face a dilemma* (P13). Participants P15 and P19 made similar comments: … *otherwise I have difficulty in functioning, or I might change jobs.* Participant P20 also made a similar point, noting that *For me, it is fundamental to have my colleagues’ support and cooperation.* Participant P16 agreed with this view.

### Parents’ wishes and attitudes

All participants agreed regarding the need for parental involvement in the decision-making process to the greatest extent possible (e.g., P14, P20): *For extremely premature babies, I let parents have their own space and views* (P12). However, participants expressed the opinion that parents could not play the key decision-making role (P3, P10, P14) because of their lack of medical literacy and their strong emotions (P3, P14), which prevent them from seeing reality clearly and cause them to make decisions that are not well-balanced. In that respect, participants noted that parents’ emotions may change over time (P10) and fathers may be less emotionally loaded than mothers (P12).

In most cases, parents may put great pressure on neonatologists (especially on empathetic neonatologists). They may express their demands or wishes either verbally, that is, through dialogue, or nonverbally, e.g., through an appealing look or facial expressions (P8). Unsurprisingly, our data analysis indicated that infants’ parents exert a great deal of pressure in the majority of cases (but not in every case), and they ask neonatologists to make every effort to keep their infant alive and functioning. For instance, participants noted that *Parents can be stress factors* (P2) … *they pushed us to intervene and do whatever is possible. And this created extreme pressure and emotional load* (P15). *We constantly intervene in a newborn’s body that has already gone through a lot, and we know it will pass away. In most cases, we do it just because it is the parents’ wish, to tell you the truth* (P16). Parents may demand that neonatologists do their utmost to save the life of their infant because of their willingness to care for and bring up the child, e.g., because it is perceived as a gift from God (despite the fact that it is disabled) (P2, P9, P19) or because of their desire to be at peace with themselves (P12). Indeed, various factors (including faint hopes, religiosity/spirituality, emotions, moral consciousness, parents’ need to touch their baby to realize that it has been on the earth as a human being, even for a short time) emerged from the data analysis as reasons why parents’ attitudes towards neonatologists caused them to exert pressure (that is, they wanted too much): *We know that the child will suffer from severe cerebral palsy and mental retardation. Nevertheless, Greek parents expect the child, whatever child this is. They will say, this is God’s decision, this is what God decided for us. They are more sentimental in this part* (P2). Furthermore, Participant P12 noted that *There are parents who tell you, I don’t care about the baby’s condition, I want you to do everything you can to save this child. So, you ought to try, you have to. Because these parents want to feel that they did everything they could for this kid* (P12).

While neonatologists do not feel as if they are explicitly prevented from acting on what they know to be right (for instance, to avoid resuscitation or to redirect the treatment provided), they may feel as if they face considerable pressure from parents to act in accordance with the parents’ demands. Unsurprisingly, empathetic neonatologists experience great emotional pressure. They put themselves in the position of parents who are experiencing a highly traumatic event. This situation is particularly prevalent for parents who have made multiple previous attempts to have a child through the use of artificial reproduction technology (P16, P18, P19), parents who want to touch their infant (who is still alive, albeit fated to die) (P19), or even parents who merely want to be at peace with themselves (to have a clear conscience) after having done everything possible for their infant (P12). In that context, Participant P14 noted that *… [The child] may be intubated, have catheters, be mechanically supported by a thousand machines, but for them, this newborn baby exists. It is alive. Let it be mechanically supported by a thousand machines. It is alive. So, they hope* (P14).

Participant P19 wondered whether keeping an infant alive even for a few hours may be of considerable importance to his or her parents since it offers them the opportunity to prepare themselves or even touch their living baby and thus to have a memory of their baby’s existence on the earth as a human being, even for a short time: *Are these few days of life important because the parents try to prepare themselves? Or is it because they want to touch the child, to feel that this baby once existed for them?* (P19) [Note, however, that as mentioned above, Participant P14 noted *How is it possible to hit a newborn continuously and catheterize it at the very moment when it is dying? Am I doing that in order to prolong its life for 1, 2, 3 days?*]. Furthermore, one participant noted that … *the parents expect a good word, which I cannot say, and with their stares and verbal and nonverbal communication, I feel as if they are pushing me psychologically* (P8). Similarly, Participant P6 noted that *“…parents are under great pressure…we know that…they try to elicit from us even a single word indicating good news about the condition of their infant* ….

As a result, neonatologists may exhibit empathy-driven emotional responses, which can serve as (soft, internal) constraints that may cause them to experience constraint distress. On the other hand, participants noted that neonatologists who exhibited interest, compassion and empathy and were viewed as doing their best to optimize the infants’ outcomes rather than simply fulfilling their operational duties under the law were more likely to gain mothers’ trust (P18).

Doctors may cause parents to become more persistent in the following situations. a) When the latter are not adequately informed or when they are overinformed: While all participants (e.g., P1, P2, P3, P9, P12, P13, P14, P15, P17, P19) expressed the view that parents should be provided with sufficient information, some participants expressed the view that parents should not be provided with overwhelming information regarding everything might be relevant to their decision: *I don’t want to have them face my dilemmas and queries. Their own load is enough* (P18). *Besides, they have no scientific grounding* (P3). b) When neonatologists invest excessive effort into the task of providing (“futile”) treatment to the infants, thus offering parents only a small amount of hope and encouraging them to develop the forlorn hope that their infant may survive (P15). c) When neonatologists show parents that they are merely doing their job: *Basically, what parents need is to show that you personally care about their child and them and that you don’t simply do your job* (P18). *The great majority of parents look you in the eyes and wish to see that you care about their child and them* (P20). Finally, d) when doctors create close emotional relationships with parents. Therefore, some participants noted that they kept parents at a distance (e.g., by speaking to them in the plural form) to avoid encouraging them to become more demanding or to prevent themselves from being involved with parents’ emotions (P17, P19). As Participant P4 noted, *The more you associate with the parents and the more you get emotionally involved with them, the higher the moral pressure is* (P4). Similarly, another participant noted that *with parents, I keep a safe distance, to tell the truth. I don’t want to be very close to them as they can be pressing and insistent. They cross the line sometimes. They try to influence your judgement while they are driven by emotion* (P19). Participants P17 and P20 expressed similar claims.

Furthermore, if parents do not take a clear stance on their infant’s treatment (e.g., because they are ill-informed) or do not share any decision-making responsibility, they do not contribute to the task of mitigating the neonatologists’ uncertainty distress. When parents show confidence in neonatologists to such an extent that they place considerable emphasis on neonatologists’ freedom to make decisions on their own, their contribution to the decision-making process is left to the discretion of the neonatologists, who “know better”. Thus, when parents offer neonatologists broad discretion to make a decision regarding resuscitation or treatment, the degree of neonatologists’ uncertainty remains high. Moreover, parents’ concerns may underlie their hesitancy towards neonatologists, which are expressed through nonverbal communication. For instance, as one participant noted, *When parents are critical, sceptical and distrustful towards me and I can see doubt in their eyes, then I feel psychologically stressed, and I try to inform them properly. I want both of them to be aware, to be part of the decision that must be made and share the load, the moral burden, the biomoral burden of the responsibility* (P13).

In summary, parents may cause neonatologists to feel constraint distress. On the other hand, parents may be supportive of the neonatologist’s decision and thus mitigate their uncertainty distress.

## Neonatologists become more resistant to moral distress over time

As a final note concerning our data analysis, it should be mentioned that according to participants, the likelihood of being able to cope with such moral (uncertainty) distress increases as their work experience increases: *We are trained to deal with the moral pressure regarding whether we have done what is proper after a certain point* (P12).

Participants P8 and P9 expressed similar claims. Moreover, the fact that participants felt morally obliged to comply with parents’ wishes rather than forced to give into those wishes due to fear of possible legal consequences (malpractice claims or lawsuits filed against them by parents) should be highlighted.

## Discussion

### Factors associated with moral distress among neonatologists

NICU directors emerged from the data analysis as key predisposing factors that are associated with constraint distress among neonatologists. In the era of shared decision-making, it is rare for NICU directors to act in a purely paternalistic fashion towards neonatologists and to impose on them or other health care providers the requirement of a burdensome treatment that they do not believe to be in the infant's best interest [7]. Despite the lack of such an apparent imposition, neonatologists may feel constrained because they value the opinion of the NICU director, who plays the role of a musical director/moderator. Furthermore, participants in this study noted that parents may put pressure on neonatologists to do their utmost to save the infant’s life either directly or by eliciting empathy. Parents “wanting too much” may be a major cause of constraint distress (moral distress in the original/strict sense of the term) among neonatologists [21]. According to the findings that emerged from our data analysis, it is difficult for neonatologists to strike a balance between the infant’s best interest and respect for parents’ autonomy in the context of EPI resuscitation, especially when they cannot “successfully set limits to parents’ requests without overriding them entirely” [8]. This difficulty may cause neonatologists to experience moral distress [8], which, however, is rarely shared [21]. Conversely, neonatologists may develop uncertainty distress when parents do not take a clear stance on the infant’s treatment or do not pressure neonatologists to do their utmost to save the infant’s life since neonatologists are nevertheless willing to fight for the minimal chance of the infant living a long life without experiencing neurodevelopmental disorders. Cavolo et al. note that neonatologists may develop moral distress “not only when they felt they were doing “too much” but also when they felt they were “not doing enough”” [8].

### Prognostic uncertainty: A major issue that is associated with moral distress among neonatologists

Moreover, prognostic uncertainty emerged as a key factor associated with moral distress in the broad sense of the term (uncertainty distress). Andaya et al. imply that prognostic uncertainty regarding the alleviation of long-term suffering (e.g., pain or disability) for neonates and their families may give rise to uncertainty distress [14]. Prognostic uncertainty (especially with regard to infants born at very low GA) is a major issue in neonatology that is associated with moral distress among neonatologists. Given the great prognostic uncertainty in this context, no reliable evidence-informed guidelines have been developed to provide recommendations intended to guide neonatologists regarding the management of infants born at very low gestational ages. Wood et al. thus rightly note that “Resuscitation care planning for extremely low gestational age neonates (ELGANs) is complex and ethically charged. Increasing survival at lower gestational ages has had a significant impact on this complexity” [13]. Accordingly, the resuscitation thresholds used by different neonatologists vary considerably and seem to be dependent on the context at hand rather than the country in question. Wilkinson et al. found a relatively narrow range of resuscitation thresholds used by different neonatologists across the UK, Sweden, and Netherlands. However, these authors found a wide range of different resuscitation thresholds among different neonatologists [15]. In addition, it is difficult to predict the short- and long-term outcomes of treatment in EPIs. Furthermore, the literature has not reached consensus regarding whether GA is an appropriate and sufficient criterion to guide resuscitation decisions [8]. It has been argued that neonatologists’ resuscitation decisions should be guided not only by GA but also by other significant factors, such as prognostic factors, parents’ values, preferences or other characteristics that can generate empathy [16]. Cavolo et al. conducted a qualitative research study and concluded that neonatologists “make resuscitation decisions based on a much more complex interplay of factors rather than on GA alone” [16].

Prognostic uncertainty is of great importance when making resuscitation decisions. The final resuscitation or treatment decision may conflict with the current guidelines, as is the case in the Netherlands and Canada [13, 17]. Wood et al. highlight evidence of moral distress among Canadian neonatologists even when treating newborns with a gestational age less than the guideline-based resuscitation threshold, that is, GA > 25 weeks [13]. These authors report evidence suggesting that “the consideration of routine resuscitation from 24 weeks and above is a more ethical approach in the current era of improved outcomes” [13]. Gestational age-based guidelines have been strongly criticized as being overly simplistic, ignoring uncertainty and disrespecting other key prognostic factors [15]. It has been argued that labelling newborns in accordance with gestational age-based guidelines may be “not only scientifically flawed, but ethically questionable” [22]. Gestational age-based guidelines for providing care to EPIs do not take into account the role of emotions [23]. Verweij et al. conducted a survey and found that “the GA-based-plus guideline which advises to take into account other prognostic factors than just GA is mostly preferred” [17]. It has been argued that decisions should be individualized/personalized [15, 17]. The individualization/personalization of care at the limit of viability should be considered independent of guidelines [17]. Verweij et al. note that in the Dutch context, the desire for “personalization or individualization of care at the limit of viability is increasing” [17]. The authors state, it can mean “to take into account other prognostic factors than GA” but also “to take into account parental wishes and values” or “to adjust the information shared in counselling to the parents being counselled” [17].

### Parental values and wishes give meaning to the prognosis

Parental values and wishes give meaning to the prognosis and should therefore be integrated into the shared decision-making process [24]. To ensure successful individualized/personalized treatment decisions in neonatology, aid should be provided to “help parents discern their own values and preferences” [17]. This claim is in line with the findings of this study. Parents with an infant in the NICU often face situations that place them at odds with their own concepts of good and their own values and beliefs. Participants in this study were opposed to the notion of providing overwhelming information to parents. This finding is in line with the conclusions of a previous study that argued against providing information to parents regarding everything that, in the opinion of the neonatologist, might be relevant to their decision. The study in question argued that “This may be overwhelming for many parents. Instead, doctors should try to discern, on a case-by-case basis, what particular parents want and need” [23]. The findings of this study are in line with this consideration. Interestingly, Lam et al. suggest the “inclusion of experienced parents of preterm infants for more effective counseling of parents in making life-and-death decisions” [25].

### Practical wisdom: An alternative tool for ethical decision making in neonatology

Furthermore, making resuscitation decisions in a very short time frame causes neonatologists’ decision uncertainty to remain very high. This finding emerged from our data analysis in the context of making decisions in the delivery room or during a shift. In cases of EPIs (for whom clinical practice guidelines do not exist) or neonatologists who are willing to fight for the minimal chance of the infant living a long life without experiencing neurodevelopmental disorders even when that approach conflicts with the current guidelines, the Aristotelean notion of practical wisdom (= *phronesis*) can serve as an alternative tool for ethical decision-making based on wisdom gained through the previous ethical decisions made by physicians. “While moral virtues enable us to achieve the end, *phronesis* makes us adopt the right means to that end” [26]. Practical wisdom (phronesis) is the virtue that allows neonatologists to make morally correct decisions and employ nudging when making shared decisions in the NICU [27]. This situation is particularly prevalent in the case of a neonatologist who must make a decision in a very short time frame (e.g., in the delivery room). Time constraints may cause considerable uncertainty distress. **“**However, the theory of *phronesis*-based medical decision-making tends to focus on individual practitioners rather than practice-based communities of physicians” [26]. Therefore, Conroy et al. offer the notion of “collective practical wisdom”, namely, a “moral debating resource” that is used as a “tool for introducing and cultivating the concept of *phronesis*”, as a starting point [26]*.* The findings of our study, particularly neonatologists’ need to seek third-party approval of their decision, highlight the value of establishing such a starting point of “collective practical wisdom” when making decisions (thereby reducing decision uncertainty distress) regarding whether to provide care for infants at the limits of viability (i.e., those born at periviable gestation). Accordingly, it should be noted that according to the participants in our study, their need to obtain the decision-making support they desire cannot be met by their colleagues. While participants felt that in their NICUs, colleagues generally supported their ethical decisions, they perceived this support as inadequate with regard to addressing their uncertainty-based psychological distress and noted that they were in need of professional psychological counselling [P2,P4,P13]. This finding is in line with the extant literature [28]. The findings of this study highlight the need to establish a starting point of “collective practical wisdom” when making decisions.

### Parental involvement in shared decision making in the NICU

The results of the systematic literature review conducted by Cavolo et al. indicate that the included authors agreed that parents should be actively involved in resuscitation decisions [29]. According to another study conducted by Cavolo et al., “neonatologists agreed on the importance of parental involvement, the degree of which depends on the EPIs’ GA” [16]. All participants in our study highlighted the importance of involving parents in the shared decision-making (SDM) process. This claim is also widely accepted in the extant literature. It should be noted that “Withholding or withdrawing intensive care for extremely preterm infants at the limits of viability with parental involvement has become more acceptable than it was 20 years ago” [30]. In addition, it has been argued that parents’ involvement in SDM helps them experience less long-term grief [31]. As resuscitation decisions for infants born at the limits/margin of viability (i.e., the “grey zone”) have increasingly become a major and ongoing challenge in clinical practice, the SDM model, which involves health care professionals and parents (as the primary stakeholders), has become the most widely accepted decision model [15, 24]. However, the SDM model is complex and difficult to apply in practice for various reasons. Below, we mention some of these reasons.

Soft medical paternalism may affect the implementation of the SDM model. It has been argued that “parents' decision should be over-ridden when in contrast with the EPI's best interest” [29]. However, this task is not always simple given that neonatologists acknowledge the fact that “there is always a certain degree of uncertainty regarding outcomes, making it difficult to overrule parents” [8]. Importantly, the infant’s best interest is difficult to determine. This notion should be considered in light of its social context. In the grey zone, stakeholders with different values who are involved in a shared decision-making process are most likely to disagree regarding the proper course of treatment. Indeed, “there are different views about where the boundaries of the gray zone should lie” [15]. However, all participants in this study emphasized the fact that they would never let parents’ preferences guide their decision-making. Neonatologists’ soft paternalistic attitudes are not inexplicable. In the United States, the model of parental autonomy prevails, whereas in France, the model of medical paternalism, according to which parents are excluded from the decision-making process, prevails [32]. It has been argued that in Greek intensive care units, medical paternalism prevails in end-of-life decisions [33]. This medical paternalism is associated with culturally conditioned attitudes regarding the value of human life [33]. Traditionally, Greek physicians have viewed the core purpose of medicine as closely related to the core values of Hippocratic professional ethics, and their intuitions are oriented towards the role of the healer [34]. This characterization is in line with what is called “Mediterranean bioethics”. Greek health care ethics are firmly entrenched in so-called “Mediterranean bioethics.” The Aristotelean ethics of virtue and friendship based on trust alongside spirituality and the sanctity of life are essential components of “Mediterranean bioethics,” which was developed by the thought of Hippocrates and great Greek philosophers and subsequently strongly influenced by the three great monotheistic Mediterranean religions (Christianity, Judaism, and Islam) [35]. This bioethics may be one of the reasons underlying not only neonatologists’ paternalistic attitudes but also their willingness to provide care for extremely premature infants and their uncertainty moral distress when facing the dilemma of whether to resuscitate these infants.

Furthermore, it should be noted that parents’ decisions do not always remain stable over time. Parents may experience less decisional regret after foregoing resuscitation than the decisional regret they would experience if their child were to survive resuscitation but suffer from severe disability [36]. This claim is in line with a finding of this study.

Moreover, the role of parents’ socioeconomic status in determining the long-term outcomes of applying the SDM model is less than clear. While “there is a documented relationship between parents’ socioeconomic status and EPIs’ neurodevelopmental outcomes” [8], in countries in which solidarity is an important value (e.g., in Switzerland), “long-term economic considerations do not interfere with decision-making at the limit of viability” [24]. This situation may (at least partly) explain the finding of a qualitative study conducted in Belgium, which suggests that neonatologists “were more interested in the impact that resuscitation decisions have on EPIs and their family rather than the impact that they might have on society at large” [8]. In that context, however, one participant in this study wondered what social welfare structure might address the needs of a person with neurodevelopmental disorders in Greece.

### Other factors influencing implementation of shared decision making (SDM) in the NICU

#### Organizational- and system-level factors

In addition, to apply SDM appropriately, we should consider the role of “organizational- and system-level factors” and examine perspectives “beyond the clinician-patient dyad” [37]. To that effect, as uncertainty is considerable in the context of the NICU, this factor should be incorporated into the SDM process. Interestingly, Berger states that “uncertainty comes in many different forms that may overlap” and that “In order to ensure that SDM can be realistically applied to real-world clinical encounters, the issue of uncertainty should be recognized and explicitly incorporated into SDM strategies” [38]. However, such an incorporation of uncertainty is not an easy task. The implementation of the SDM model in the context of the NICU against a backdrop of great uncertainty may give rise to a variety of challenges, advantages and limitations in the context of emerging technologies [39]. In these circumstances, it seems to be extremely difficult for neonatologists, who are compelled to make decisions in a short time frame, to implement the SDM model appropriately. Making decisions in a short time frame is another key factor that has a profound negative impact on the successful implementation of SDM in the context of neonatology. The association between shared decision-making and time pressure remains understudied [40].

#### Effective communication

In addition, neonatologists’ lack of the specific communication skills necessary to practice SDM properly against a backdrop of great uncertainty is another key factor that undermines the successful implementation of the SDM model in real-world clinical practice [40, 41]. Specific communication skills are required to successfully implement the SDM model in real-world clinical practice. However, even in developed countries, ethicists do not engage in postmedical school education. Limitations in terms of physician communication may unwittingly undermine the successful implementation of the SDM model in real-world clinical practice [41]. As complex decisions regarding periviable interventions have far-reaching consequences, an experienced neonatology team should be involved in SDM, and trust and effective communication among all involved stakeholders is necessary in this context [37].

#### Best interest and quality of life

Ultimately and most importantly, the fact that in the context of neonatology, the best interest and quality of life of the infant are difficult to determine emerged from our data analysis. This situation can make it even more difficult to implement SDM properly. Stakeholders’ judgements are often based on their personal views and evaluations rather than facts. Indeed, neonatologists can attempt resuscitation when it is considered to be in the infant's best interest [42]. EPIs’ best interests are “not closely related to survival rates or disability” [42]. EPIs “are systematically devalued, in comparison with older patients whose outcomes are the same or worse” [42].

### Who is the ultimate decision-maker?

In case of disagreement among the stakeholders involved in an SDM process, a crucial question is who should be the ultimate decision-maker. Importantly, Cavolo et al., in their qualitative study in Belgium, concluded that neonatologists “involve parents differently depending on the EPI’s GA” [16]. Accordingly, Cavolo et al. put it best by noting that in the shared decision process, “the weight of decision power bends on parents or physicians depending on GA” due to inappropriate implementation of the model in real-world practice [16]. In a study conducted by Bucher et al., a large percentage of respondents expressed the view that Institutional Ethics Committees (IECs) should be the ultimate decision-makers, with a large majority of Swiss laypeople believing that parents should be the ultimate decision-makers [24]. Indeed, the IEC “may serve as a valuable resource for staff and parents dealing with difficult ethical decisions” [43]. IECs that are available in hospitals with NICUs may serve as the ultimate decision-makers, with neonatologists being aware of “when the committee might be helpful, or how it functions” [43].

At any rate, it is important to bear in mind the fact that SDM is a multifactorial process that has not yet been fully explored. While the SDM process has been studied extensively, it remains unknown and can be improved [40]. At present, however, this method cannot be “equally implemented in consultations with every patient” [40]. Neonatologists who are aware of the inappropriate application of SDM and who disagree with the parents regarding the action that should be taken as part of the infant’s treatment may experience considerable uncertainty distress, which may be one more reason they distance themselves from parents.

### Limitations

This study should be interpreted in light of certain limitations. Ten of the total of twenty participants had 1–3 years of professional experience as neonatologists working in the NICU; that is, they had limited experience with challenging cases pertaining to the limits of viability. Furthermore, only five out of twenty participants were female. Future qualitative studies may evaluate possible differences in the perceptions of neonatologists according to work experience and gender. Finally, participants were not asked to check the consistency between their intentions and the results obtained by the researchers. This fact limits the reliability of the study in terms of confirmability.

## Conclusion

We concluded that neonatologists’ moral distress should be conceptualized in the broad sense of the term and is closely associated with multiple predisposing factors. Such distress is greatly affected by interpersonal relationships. A variety of distinct themes and subthemes emerged from the analysis of the interview data. Neonatologists face moral uncertainty**.** Furthermore, they prioritize their traditional (Hippocratic) role as healers**.** Importantly, neonatologists seek third-party support for their decisions to reduce their decision uncertainty. In addition, based on the analysis of the interview data, multiple predisposing factors that foster and facilitate neonatologists’ moral distress emerged, as did multiple predisposing factors that are sometimes associated with neonatologists’ constraint distress and sometimes associated with their uncertainty distress. The predisposing factors that foster and facilitate neonatologists’ moral distress thus identified include the lack of previous experience on the part of neonatologists, the lack of clear and adequate clinical practice guidelines/recommendations/protocols, the scarcity of health care resources, the fact that in the context of neonatology, the best interest and quality of life of the infant are difficult to determine, and the need to make decisions in a short time frame. NICU directors, neonatologists’ colleagues working in the same NICU and parental wishes and attitudes were identified as predisposing factors that are sometimes associated with neonatologists’ constraint distress and sometimes associated with their uncertainty distress. Ultimately, neonatologists become more resistant to moral distress over time. All the themes and subthemes that emerged from the analysis of the interview data were largely consistent with the extant literature. However, we identified some nuances that are of practical importance. Further research might support the needs to establish institutional ethics committees in every health care setting and to make neonatologists aware of those committees. Ultimately, the results of this study may serve as a starting point for future research.

## Data Availability

The transcripts of the full interviews that were collected and qualitatively analysed in the current study are not available for reasons of confidentiality. The redacted transcripts that were used and analysed as part of the current study can be made available by the corresponding author upon reasonable request.

## Abbreviations

NICU: Neonatal intensive care unit
SDM: Shared decision making
GA: Gestational age
EPI: Extremely premature infant
