# Correction: Moral distress among neonatologists working in neonatal intensive care units in Greece: a qualitative study

**DOI:** 10.1186/s12887-024-04802-2

**Published:** 2024-05-06

**Authors:** Maria Deligianni, Polychronis Voultsos, Maria K. Tzitiridou‑Chatzopoulou, Vasiliki Drosou‑Agakidou, Vasileios Tarlatzis

**Affiliations:** 1 Laboratory of Forensic Medicine & Toxicology (Division: Medical Law and Ethics), School of Medicine, Faculty of Health Sciences, Aristotle University of Thessaloniki, University Campus, Thessaloniki, GR 54124 Greece; 2 Midwifery Department, School of Healthcare Sciences, University of Western Macedonia (Greece), Ikaron 3, Kozani, GR 50100 Greece; 3 1st Department of Neonatology, School of Medicine, Faculty of Health Sciences, Aristotle University of Thessaloniki, University Campus, Thessaloniki, GR 54124 Greece; 4 1st Department of Obstetrics and Gynaecology, School of Medicine, Faculty of Health Sciences, Aristotle University of Thessaloniki, University Campus, Thessaloniki, GR 54124 Greece

**Correction:** ***BMC Pediatr*** **23, 114 (2023)**

10.1186/s12887-023-03918-1

Following the publication of the original article [1], the authors omitted to include the funder in the Funding statement. The online version of this article has been updated to reflect the correct declarations. The authors disclosed receipt of financial support for the research, authorship, and/or publication of this article. Therefore, the revised version of the Funding section paragraph should read as follows: Funding: “This research was co-financed by Greece and the European Union (European Social Fund- ESF) through the Operational Programme «Human Resources Development, Education and Lifelong Learning 2014–2020» in the context of the project “Qualitative research on doctors’ moral distress and parents’ moral distress and moral schism in neonatal intensive care units” (MIS 5047889).” The authors apologise for this error and any confusion it may have caused.
